# Computational approaches for RNA structure prediction and design

**DOI:** 10.1016/j.xcrp.2026.103097

**Published:** 2026-02-18

**Authors:** Yuki Kagaya, Boyuan Liu, Daisuke Kihara

**Affiliations:** 1 Department of Biological Sciences, Purdue University, West Lafayette, IN 47907, USA; 2 Department of Computer Science, Purdue University, West Lafayette, IN 47907, USA

## Abstract

Determining the three-dimensional (3D) structure of RNA is crucial for understanding its diverse biological functions. The field of computational RNA structure prediction has recently been transformed by deep learning, which has dramatically improved accuracy beyond that of conventional homology- and *de novo* modeling approaches. This article overviews these advancements. We first summarize the principles of foundational conventional approaches before detailing the current state-of-the-art deep-learning-based approaches. Deep-learning-based approaches are categorized into strategies that leverage multiple sequence alignments (MSAs), recent MSA-free methods that rely on single sequences, and emerging generalist models that can predict entire heterogenic biomolecular complexes. Furthermore, we discuss how these predictive breakthroughs are accelerating the field of RNA design. Finally, we outline the current challenges and future directions for computational RNA structural biology.

## INTRODUCTION

Determining the three-dimensional (3D) structure of biological macromolecules remains a central theme in biophysics and bioinformatics, as structure is inextricably linked to function. For decades, the prediction of protein structure from its primary amino acid sequence was considered a grand challenge. Computational efforts to address this problem largely relied on strategies such as homology modeling,^1^ which uses experimentally known structures as scaffolds, and the analysis of coevolutionary information from multiple sequence alignments (MSAs) to infer residue contacts.^2^ However, the landscape was dramatically reshaped by the recent revolution in artificial intelligence. Deep-learning methods, most notably AlphaFold,^3^ have profoundly advanced our predictive capabilities, achieving remarkable accuracy across a wide range of proteins. While this transformative progress has opened new avenues in biological research, it has also highlighted the field’s remaining challenges, such as the prediction of protein dynamics,^4^ thereby broadening the scope of computational structural biology. Within this expanding horizon, a new grand challenge has come into sharp focus, namely the prediction of RNA 3D structure. This is particularly critical for the vast class of noncoding RNAs (ncRNAs), which are not translated into proteins but function directly. This class includes a wide array of functionally critical molecules, such as ribosomal RNAs (rRNAs),^5^ which form the catalytic core of the ribosome; microRNAs (miRNAs),^6,7^ which regulate gene expression; and riboswitches, which act as metabolic sensors.^8^ For these molecules, accurately predicting their intricate 3D folds is the key to understanding their biological roles, presenting a formidable set of challenges that now demand the community’s full attention.

The advancement of predictive methods is periodically evaluated by rigorous and objective assessment in the bioinformatics community. In protein structure prediction, this role has been fulfilled for decades by the Critical Assessment of Techniques for Protein Structure Prediction (CASP) experiment. Through a biennial blind challenge in which participants predict structures before they are publicly released, CASP has served as the gold standard for unbiased evaluation, stimulating competition and driving methodological innovation. In the RNA-structure-prediction community, RNA-Puzzles^9–13^ has long served as the principal platform for community-wide blind assessments. Unlike CASP’s fixed biennial schedule, RNA-Puzzles operates on a rolling format, releasing new targets as they become available. The growing importance of this field has recently been underscored by the inclusion of RNA as a formal category within the main CASP framework from CASP15 held in 2022. This development provides an opportunity to transfer the methodological advances achieved in protein structure prediction to the RNA domain, accelerating the maturation of RNA structural bioinformatics. In the first-ever RNA category in CASP15,^14^ the top-performing methods were limited to conventional approaches and deep-learning methods were scarcely represented, whereas the most recent CASP16 round^15^ in 2024 saw a dramatic shift, with deep-learning-based methods rapidly emerging as central and highly competitive players.

To rigorously evaluate the quality of predicted RNA structures, several metrics are commonly employed in community challenges such as RNA-Puzzles and CASP. Similar to protein structure assessment, the root-mean-square deviation (RMSD) serves as a standard measure for global coordinate accuracy. The template modeling (TM) score is another widely used metric that addresses the limitations of RMSD, such as its sensitivity to outliers and its dependence on RNA length.^16^ For RNA assessment, TM score typically utilizes C3′ atoms by default and employs specifically customized parameters for nucleic acids. In addition to these global metrics, the local distance difference test (lDDT) is used to evaluate the accuracy of local structural coordinates.^17^ Furthermore, interaction network fidelity (INF) is used as an RNA-specific metric.^18^ Since base pairing and stacking are fundamental to RNA function, INF is frequently used to assess how accurately these interactions are predicted within the model.

Driven by the recent advance and success of deep learning, this review presents an overview of the computational approaches for RNA 3D structure prediction and RNA design. It begins by summarizing the principles of conventional prediction methods, which established the theoretical foundations of the field and continue to provide valuable insights. The main focus of the review is then directed toward state-of-the-art deep-learning approaches, highlighting their diverse architecture, the input features they utilize, and the key innovations behind their recent achievements. Finally, the review considers the broader impact of these developments on applications such as RNA design, discussing how access to highly accurate structural models can accelerate the rational design of RNA-based therapeutics and functional molecules and contribute to the future of RNA bioinformatics. The methods discussed in this review are summarized in Table 1.

## CONVENTIONAL APPROACHES

First, we discuss conventional methods for RNA structure prediction. They can be roughly classified into two approaches: homology modeling, which use known structures as scaffold of modeling; and methods that use a coarse-grained structure model with statistical or physical energy functions. Before the recent rise of deep learning, the landscape of computational RNA structure prediction was defined by methods grounded in biophysical principles and evolutionary information. Historically, efforts to predict the 3D structure of RNA were often preceded by the prediction of its secondary structure.^19^ The secondary structure of RNA is regarded as a 2D map that describes the pattern of canonical base pairs, such as A-U and G-C, within the sequence. This 2D blueprint is informative for modeling because it defines the core architectural elements such as stems, loops, and junctions, which provide the fundamental scaffold for the final 3D folding. Consequently, many early 3D-structure prediction methods were designed to use secondary-structure information, either experimentally obtained or hypothesized based on sequence similarity or computational prediction, as a primary input.^20^ This information served as a guide to simplify the complex task of arranging atoms in 3D space. Within this framework, two major strategies became dominant, namely homology modeling and *de novo* modeling. This section briefly outlines the core principles, strengths, and inherent limitations of these foundational approaches.

### Homology modeling

Homology modeling, also known as comparative or template-based modeling, represents the most direct and often the most reliable strategy for 3D structure prediction when templates are applicable. The central idea of this strategy is that, if a target RNA sequence shares significant similarity with an RNA whose structure has already been experimentally determined, it is highly probable that it adopts a similar 3D fold. The modeling process typically involves searching experimentally determined structures in the database, for instance the Protein Data Bank (PDB),^21,22^ for a suitable template, aligning the target sequence to the template’s sequence and then transferring the coordinates of the aligned regions. The final step involves building the coordinates for any unaligned regions, such as insertions or deletions, and refining the entire model.

While homology modeling for proteins has long been supported by automated servers, the development of tools for RNA was behind. For earlier years, the process remained largely manual or semi-automated, with experts using their knowledge and interactive programs such as S2S and Assemble to perform the modeling.^23,24^ To automate this complex workflow, fully automated software has been developed to facilitate this type of homology modeling. ModeRNA^25^ is a representative example of a comparative modeling program that automates many of these steps. It allows users to build a model of a target RNA based on a given template structure and sequence alignment provided by the user. The method can also handle non-canonical base pairs and chemically modified nucleotides. ModeRNA can consider multiple structures as templates and is capable of re-constructing missing segments, such as loops, by utilizing existing or user-defined fragment libraries. RNAbuilder^26^ is another example. Although it shares many features with ModeRNA, such as the ability to incorporate multiple structural templates, its distinguishing characteristic is a coarse-grained simulation-based assembly process guided by user-defined constraints, including secondary-structure information.

In cases where sequence similarity is low, threading-based approaches can identify structural templates by considering both sequence and structure information. Foldalign^27^ aligns RNA sequences by simultaneously considering sequence similarity and potential secondary structures. It produces biologically plausible alignments by combining a scoring system for structural stability with sequence similarity. LocARNA^28^ provides a more efficient alternative by utilizing pre-calculated base-pairing probabilities. This probabilistic approach allows it to identify structural templates in large-scale genome-wide analyses. Unlike the dynamic programming used by these tools, LaRA^29^ utilizes integer linear programming to formulate the alignment as an optimization problem. This mathematical framework enables the alignment of complex structural elements such as pseudoknots. These diverse strategies allow us to find structural templates even for RNA sequences that have significantly diverged over time.

Despite the strengths of homology modeling, the applicability of these methods is fundamentally constrained by the availability of suitable templates. The number of unique RNA structural families with experimentally determined structures remains relatively small.^30^ As a result, this approach cannot be applied for a large proportion of RNA sequences, particularly those with novel folds.

### *De novo* modeling approaches

The *de novo*, or *ab initio*, modeling approach represents another major paradigm in structure prediction. It aims to predict the 3D structure of RNA from sequence alone, without relying on any overall structural template. Conventional methods in this category treat structure prediction as an optimization problem that seeks the lowest-energy conformation within a huge search space. This task involves two fundamental challenges. The first is the sampling problem, which concerns how to efficiently explore the astronomical number of possible 3D conformations. The second is the scoring problem, which concerns how to accurately evaluate the free energy of a given conformation in order to correctly identify the native state from sampled decoys.

Energy functions are essential for both guiding the search for stable structures during sampling and ranking final candidate structures to identify the native-like fold. Knowledge-based potentials are the most widely used scoring functions in this field. These are developed by analyzing structural patterns stored in PDB. The fundamental principle is that structural arrangements frequently observed in known RNA structures are considered more stable and are given better scores. While these potentials share this common principle, they evaluate various geometric features. For example, RNA Atlas of Structure Probing (RASP),^31^ Distance-scaled Finite Ideal-gas Reference state (DFIRE-RNA),^32^ and rsRNASP^33^ utilize scores based on the distances between atom pairs. The Rosetta energy function used by the Fragment Assembly of RNA with Full-Atom Refinement (FARFAR2)^34^ combines statistical preferences with terms for physical interactions such as hydrogen bonding. In contrast, 3dRNAscore^35^ and the all-atom potential BRiQ^36^ incorporate orientations and backbone angles to represent structural geometry more accurately. It is also common to perform coarse-grained modeling in RNA structure prediction. Specific statistical potentials have been developed for these simplified representations, including the energy functions used in SimRNA^37^ and IsRNA.^38^ Collectively, these diverse scoring methods provide an effective balance of accuracy and computational speed required for evaluating large numbers of candidate structures.

Various strategies have been developed to address these challenges. To tackle the sampling problem, a widely adopted approach is fragment-based assembly. This strategy constructs RNA models by assembling structural fragments extracted from databases of experimentally determined RNA structures, significantly narrowing the conformational search space. Various methods implement this strategy. For instance, RNAComposer^39^ builds 3D models by piecing together fragments from an internal database that correspond to the elements of a user-provided secondary structure. Vfold3D^40^ constructs models in a similar fashion, breaking down a predicted secondary structure into common motifs (e.g., hairpin loops, junctions) and assembling their corresponding 3D templates. The FARFAR2 method,^34^ implemented in the Rosetta framework, also uses this principle by assembling small, overlapping structural fragments, guiding the process with a knowledge-based Rosetta energy function.

To reduce the complexity of the conformational space, a simplified representation of the RNA structure is usually used in the methods of this category. SimRNA^37^ models each nucleotide using five beads and explores conformational space through extensive Monte Carlo simulations guided by a statistical potential. Another approach, IsRNA,^38,41^ also uses a coarse-grained representation with four or five beads per nucleotide and employs a customized molecular dynamics method for conformational sampling. RNA Junction Prediction (RNAJP)^42^ integrates a nucleotide-level coarse-grained model with a helix-level coarse-grained representation, placing particular emphasis on accurately modeling multi-way junctions. Predicting the 3D topology of helices at these junctions remains a major challenge in RNA 3D structure prediction. To overcome this difficulty, RNAJP employs an enhanced sampling strategy that enables comprehensive, global exploration of helix arrangements around junctions. At last, we mention Backbone Rotameric and Quantum-mechanical-energy-scaled base-base knowledge-based potential (BRiQ),^36^ an all-atom, knowledge-based statistical potential designed to accurately evaluate RNA base-pairing and -stacking interactions. BRiQ does not generate structures but evaluates structure models. In CASP15 held in 2024, the developers’ group (AIchemy_RNA2) used BRiQ in multiple stages of modeling, including guiding Monte Carlo sampling to generate models, selecting, and refining models,^43^ achieving the top performance in the DNA/RNA category.

These *de novo* methods are able to predict entirely novel folds, but their accuracy is often constrained by the difficulty of achieving sufficient sampling and by the inherent limitations of the accuracy of those energy functions, particularly for larger and more complex RNA molecules.

### Hybrid approaches

In practice, researchers often employ hybrid approaches, combining different methods in a pipeline where expert knowledge and intuition guide the selection and tuning of tools and inputs. The top competitors of a recent RNA-Puzzles challenge illustrate this trend, which often involves significant manual intervention.^13^ For example, the Das group augmented their primary fragment-assembly method (FARFAR2^34^) with a machine-learning-based scoring function (Atomic Rotationally Equivariant Scorer [ARES]^44^), where the final models were often selected after manual inspection and refinement of secondary-structure input. The Bujnicki group provides another compelling example, effectively combining template-based modeling with ModeRNA for targets that have good templates, *de novo* folding simulations with SimRNA, and a final refinement step using Quick Refinement of Nucleic Acid Structure (QRNAS),^45^ a workflow also guided by expert decision.

Compared to these semi-manual combinations, Vfold^46^ is an approach that automated the process. It initiates the process with Vfold2D^47^ to predict a secondary structure, which then serves as an input of later steps. A 3D model is constructed by assembling structural motifs using Vfold3D^40^ and VfoldLA^48^ (a loop-template-based method). The final structure is minimized to resolve steric clashes and optimize geometry.

By integrating secondary-structure prediction, template-based motif assembly, and energy-based refinement, these hybrid strategies represent pragmatic attempts to build the most accurate models possible from all available information. Although these conventional strategies have laid an essential foundation for the field, persistent challenges in accuracy, scalability, and the prediction of novel topologies have underscored the need for more powerful methods.

## RNA STRUCTURE PREDICTION METHODS USING DEEP LEARNING

Similar to the field of protein structure prediction, the advance of deep learning has transformed RNA structure prediction. In contrast to the conventional approaches discussed in the previous section, deep-learning approaches enable models to learn complex spatial patterns directly from structural data. This made it possible to achieve levels of accuracy and generalization that were previously unattainable.

**Figure 1.**
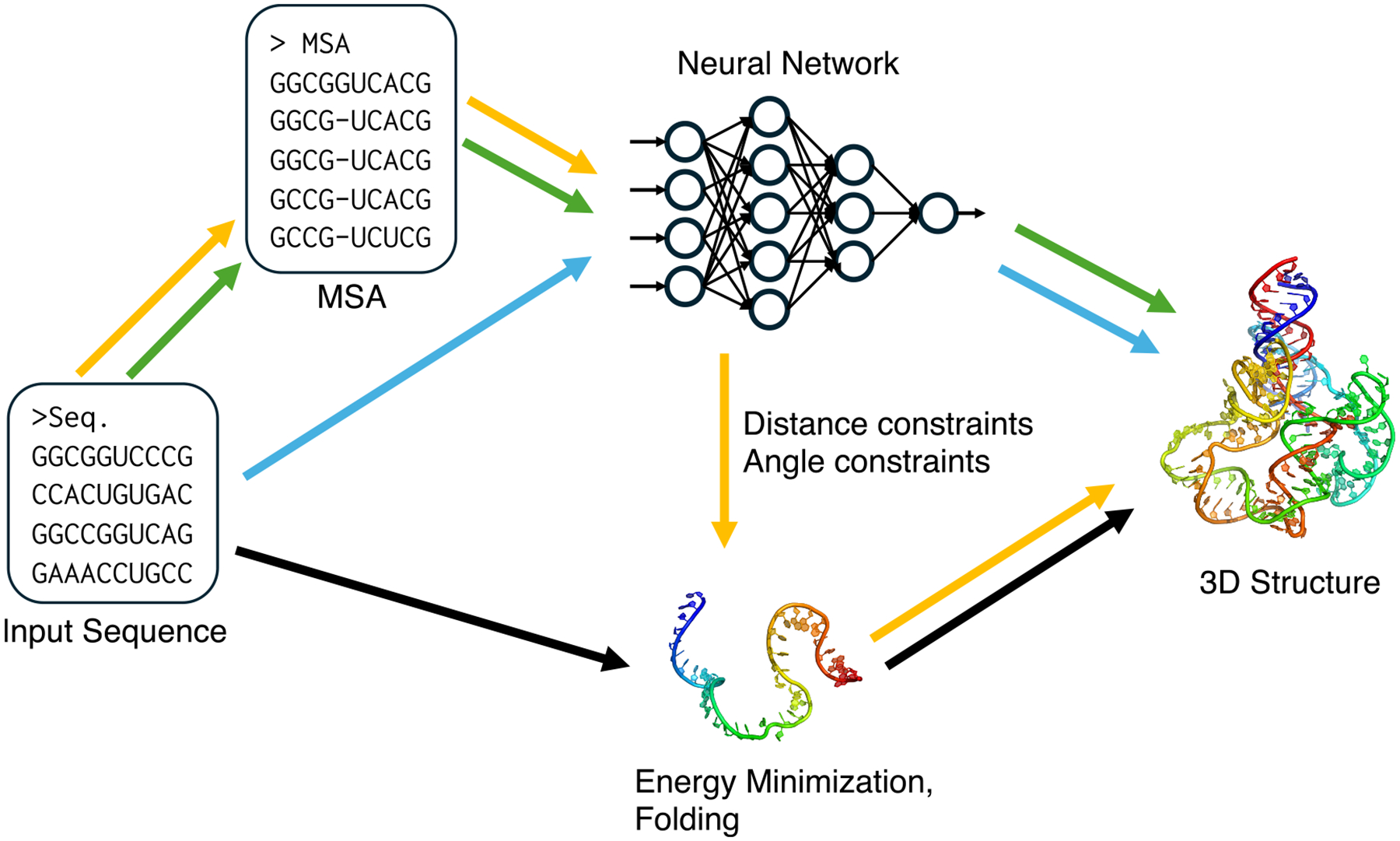
Overview of major computational approaches for RNA 3D structure prediction Four distinct paradigms are illustrated. Black arrows, conventional *de novo* folding predicts structures directly from a sequence via energy minimization. Yellow arrows, two-stage deep-learning methods use a neural network to infer geometric constraints from sequence or MSA, which then guide a separate folding algorithm. Green arrows, MSA-based end-to-end deep-learning methods directly output the final 3D structure from the input features within an integrated neural network. Blue arrows, MSA-free end-to-end deep-learning methods directly output the final 3D structure from the input sequence only using neural network.

Many recent approaches to RNA 3D structure prediction have adopted deep-learning methods originally developed for protein structure prediction. These efforts can be broadly categorized according to their input representations and modeling paradigms. One major line of development leverages MSAs to extract coevolutionary signals, which are powerful indicators of base pairing and a higher-order contact. Another emerging direction employs large language models (LLMs) trained on massive RNA sequences without structure, which attempt to capture intrinsic structural regularities without the need for explicit MSA construction. Below, we discuss these two categories of deep-learning-based approaches. The conceptual differences between the major deep-learning paradigms are illustrated in Figure 1.

Deep-learning methods for both proteins and RNA share several fundamental technical strategies. Most recent models utilize transformer-based architectures to extract meaningful patterns from sequence and MSA data. These modules are designed to be highly effective at capturing contact between distant residues or nucleotides, and a loss term for residue/nucleotide distance prediction is commonly used as a term in a loss function. AlphaFold2 and later architectures include networks that predict translation and rotation vectors for each residue to generate full-atom coordinates. For supervision during training, structural losses such as the frame aligned point error (FAPE), which was introduced by AlphaFold2, are widely used to evaluate the accuracy of predicted coordinates. Such networks also possess the ability to estimate the confidence level of the predicted models, which is also represented as a loss term.

Applying these architectures specifically to RNA requires fundamental modifications due to its unique biophysical properties. First, the main mechanism that stabilizes the structure differs between the two molecules. While hydrogen bonds along the peptide backbone serve a major factor for structure determination in protein structures, RNA folding depends heavily on base-pairing and base-stacking interactions. Therefore, networks must be designed to prioritize interactions around the bases. Second, RNA possesses a higher number of torsional degrees of freedom per residue compared to proteins. In amino acids, the primary degrees of freedom reside in the side chains. In contrast, these degrees of freedom are found within the backbone for RNA. This characteristic requires the network to predict a larger set of angles and results in much greater overall flexibility. Third, the use of structural templates follows different strategies. Protein models frequently incorporate information from known structural templates to guide the modeling process. However, this approach is less feasible for RNA because similar structures are not yet sufficiently accumulated in experimental databases. Instead, many RNA models utilize secondary-structure predictions as an alternative input. This strategy is effective because modern secondary-structure prediction methods are relatively accurate and provide an essential scaffold for building 3D folds.

### MSA-based two-stage methods

Many deep-learning models for RNA 3D structure prediction adopted a two-stage strategy, which has two separate components for prediction network and a folding algorithm. In this framework, a neural network first predicts key geometric features of the target RNA, such as inter-nucleotide distances, orientations, or torsion angles, from coevolutionary features derived from MSAs. These predicted geometric restraints are then used as constraints or energy terms in a separate folding process, where optimization algorithms reconstruct the most probable 3D structure. The two-stage approach has been also popular for protein structure prediction^49–53^ before the appearance of Alphafold2, the first end-to-end approach.^3^ This approach corresponds to the yellow arrows in Figure 1.

One of the earliest methods in this category is DeepFoldRNA.^54^ This model takes both the MSA and secondary structure as inputs and embeds them through a series of self-attention layers of deep neural network. Its architecture comprises two modules called MSA Transformer and Sequence Transformer, forming a dual-track structure similar to that used in the Evoformer module of AlphaFold2. From these embeddings, the network predicts coarse-grained backbone dihedral angles as well as distance and orientation restraints between nucleotide pairs. The final 3D structure is obtained by minimizing energy functions derived from these restraints using the L-BFGS optimization algorithm.

Another example is trRosettaRNA,^55^ which extends the successful trRosetta framework^56,57^ developed for proteins to RNA. Similar to DeepFoldRNA, trRosettaRNA takes the MSA and secondary structure as inputs and encodes them using a transformer-based network module called RNAformer. The model predicts coarse-grained backbone hinge and dihedral angles along with multiple distance and orientation distributions between nucleotide pairs. These predicted geometric potentials are then incorporated into PyRosetta^58^ as additional potential terms to guide structure folding and refinement, which is the same strategy used in trRosetta for proteins. trRosettaRNA achieved strong performance in benchmarks such as RNA-Puzzles and CASP15.

These non-end-to-end approaches have both strengths and weaknesses. One of the strengths is the interpretability of the data. The output from the network, such as angles and distances, is human-understandable information, which can be evaluated by comparing it with experimental data or other prior knowledge. Furthermore, because these predicted data are used together with knowledge-based energy functions during the folding stage, the resulting models are expected to be more stable and closer to the actual laws of physics. On the other hand, there are also weaknesses. The structure calculation is a computationally intensive process. Even with the aid of constraints derived from predictions, significant computational resources are required to achieve good structural predictions for longer RNAs. Another issue is that, because the two stages are inherently separate modules, a valid structure cannot be generated if the constraints are self-contradictory, such as distance constraints that violate the triangle inequality. Additionally, since it is impossible to utilize errors derived from the final 3D structure during the network’s training, the network cannot learn the features that are important for the accurate final 3D structure.

### MSA-based end-to-end methods

MSA provides important information about base-pair interactions. It is known that the accuracy of prediction methods is highly dependent on the depth of the MSA, with performance often declining when coevolutionary information is limited.^15^ The workflow shown in green arrows in Figure 1 represents end-to-end architectures where the network is responsible for the entire folding process, directly outputting atomic coordinates from MSAs built from the query sequence. Building such architectures has long been a difficult challenge even for proteins because of the extremely high degrees of freedom associated with all-atom 3D coordinates and also because the network becomes more complicated, which is difficult to train. In the case of AlphaFold, the first version^49^ adopted a two-stage approach, but AlphaFold2 introduced an end-to-end network, which used a structure-building module that has a new attention mechanism called invariant point attention (IPA). The structure module predicts translation and rotation for each of the backbone frames and reconstructs all atoms by predicting the torsion angles of the main and side chains. This approach greatly reduces the degrees of freedom compared to directly predicting all atomic coordinates. RNA end-to-end architectures generally adapt this concept by modifying the structure module for nucleotides.

**Figure 2.**
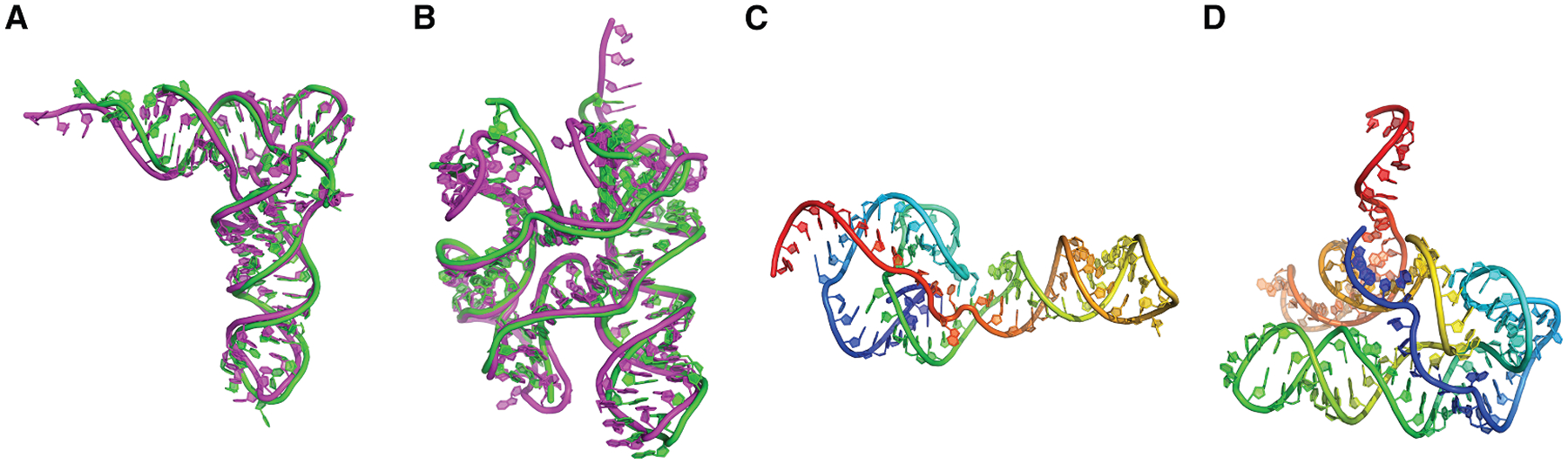
Examples of predicted RNA structures by NuFold (A and B) Predicted structures (magenta) are superimposed to experimentally determined structures (green). (A) *Escherichia coli* tRNA-Glu (PDB: 2DER chain C). Length: 76 nt. RMSD: 2.89 Å. (B) *Caldanaerobacter subterraneus* ydaO riboswitch (PDB: 4QLM chain A). Length: 125 nt. RMSD: 3.55 Å. (C and D) Examples of predictions for RNAs that do not have experimentally determined structures yet. They are stored in the NuFold Database (https://nufold.kiharalab.org). Structures are colored from blue to red for the direction of 5′ end to 3′ end. (C) Hepatitis D virus (HDV) ribozyme (RNAcentral: URS00006C1D09. Rfam family: RF00094). Length: 89 nt. pLDDT (confidence score): 90.5. (D) *Nocardia brasiliensis S-adenosyl-L-methionine* (SAM) riboswitch (RNAcentral: URS0000C049C5. Rfam family: RF00634). Length: 111 nt. pLDDT: 81.3.

The first example of such an approach is NuFold.^59^ Similar to AlphaFold2, NuFold employs both an Evoformer and a structure module. The inputs are MSAs and predicted secondary structures, where the secondary structure serves as an alternative to template information used in AlphaFold2 network. In the structure module of NuFold, the backbone frame is defined using C1′, O4′, C2′, and N1/N9 atoms around the ribose ring and base. The module predicts nine torsion angles per nucleotide, one for base and others for backbone, allowing flexible and accurate representation of base conformations. This internal representation, termed the flexible nucleobase center representation, was introduced to effectively model RNA-specific base pairing and stacking interactions. NuFold also adopted mechanisms such as recycling, in which the outputs of previous iterations are fed back into the network to refine predictions iteratively, as well as the predicted LDDT (pLDDT) score for per-nucleotide confidence estimation. With these innovations, NuFold achieves competitive global performance and high local accuracy compared to other state-of-the-art methods. In Figure 2, four examples of predicted RNA structures by NuFold are shown. The latter two examples correspond to RNA structure predictions for which no experimentally resolved structures are available. NuFold produced high-confidence predictions, with pLDDT scores of 90.5 and 81.3 for the models shown in Figures 2C and 2D, respectively.

Another representative model in this category is RhoFold+,^60^ which also adopts an end-to-end design leveraging MSAs. RhoFold+ shares architectural similarities with AlphaFold2 but differs in its preprocessing of MSA inputs using an RNA language model called RNA-FM.^61^ RNA-FM is a pretrained RNA foundation model that learns general sequence contextual patterns from large-scale RNA sequence data. This implicit knowledge complements the explicit coevolutionary information in the MSA, improving prediction accuracy particularly when MSA depth is limited. The RNA-FM-processed features are refined by the Rhoformer, a transformer network similar to AlphaFold2’s Evoformer, and the final 3D coordinates are produced by the structure module. The backbone frame is defined along the C4′, C1′, and N1/N9 atoms, and the model reconstructs full-atom coordinates by predicting four torsion angles per nucleotide. In benchmarks, the integration of RNA-FM substantially improved the model’s performance, enabling RhoFold+ to outperform competing methods across many targets.

### MSA-free methods

In contrast to the methods described in the previous sections, which rely on coevolutionary information derived from MSAs, this section focuses on MSA-free approaches for RNA structure prediction. Conventional *ab initio* methods are essentially single-sequence approaches because they do not rely on MSA information. Their accuracy has been shown to be comparable to that of deep-learning models in scenarios where explicit MSA data for the target RNA is unavailable. While MSA-based methods have achieved high accuracy, just as the most advanced protein structure predictors still depend on MSAs, this approach faces several limitations. First, constructing high-quality MSAs is difficult for novel RNA families with few detectable homologs, as well as for *de novo* designed or synthetic RNAs. Second, generating MSAs requires extensive sequence database searches, which adds substantial computational costs during inference. To overcome these challenges, recent studies have explored predicting RNA 3D structures directly from single sequences, bypassing the need for explicit coevolutionary features. These approaches aim to capture structural constraints through alternative representations, such as learned embeddings from large-scale RNA language models. These models may indirectly leverage evolutionary information learned during the training phase. Nevertheless, they eliminate the need for explicit MSA construction during inference. This allows the models to bypass the most time-consuming part of the prediction process.

DRfold^62^ demonstrated high accuracy without relying on MSAs at a time when deep-learning-based prediction methods primarily used MSA as input. The inputs required by DRfold consist of a single RNA sequence and its predicted secondary structure obtained from external tools such as PETfold^63^ or RNA-fold.^64^ The architecture of DRfold integrates the principles of end-to-end and non-end-to-end methods into a two-pipeline architecture. The first pipeline, called the structure module, performs an end-to-end approach by leveraging the IPA module to directly predict the local coordinate system for each nucleotide in 3D space, which is defined by a rigid body formed from the P, C4′, and N atoms. The second pipeline, called geometric module, implements a non-end-to-end approach by predicting pairwise nucleotide distances and torsion angles as probabilistic distributions. The final structure is constructed by integrating the outputs of these two modules into a hybrid energy potential and optimizing it using gradient-based optimization algorithms such as Limited-memory Broyden-Fletcher-Goldfarb-Shanno (L-BFGS) algorithm.

The successor, DRfold2,^65^ further develops the core concept of DRfold while completely redesigning the architecture. DRfold2 requires only a single RNA sequence as input, which is first processed by a newly developed pretrained RNA large language model called the RNA Composite Language Model (RCLM). Unlike conventional language models that predict single masked nucleotides, RCLM employs a learning strategy known as composite likelihood maximization, which considers not only the probability of a single masked nucleotide but also the joint probability of nucleotide pairs. This strategy enables the model to self-supervise and capture patterns of coevolutionary signals. The output of RCLM is further processed by RNA transformer blocks, which are similar to the Evoformer, and the denoising RNA structure module to generate the final all-atom structure. The resulting structures are then ranked and refined using the Clustering, Scoring, Optimization, and Refinement (CSOR) protocol, which consists of clustering, scoring, optimization, and refinement steps.

### Generalized macromolecule prediction methods

At the end of the RNA structure prediction methods using deep learning section, we mention recent methods that are capable of predicting the structures of proteins, DNA/RNA, and their complexes within a single framework.

Examplesof this trend include the RoseTTAFold variants, as well as AlphaFold3^66^ and its derivatives. RoseTTAFold^67^ was originally developed for protein structure prediction. Its distinctive architecture features a “3D track” equipped with an SE(3)-Transformer, in addition to a 1D and 2D track similar to that of AlphaFold2 Evoformer. The subsequently released RoseTTAFoldNA^68^ inherits these fundamental features and is extended to model both proteins and nucleic acids simultaneously. Unlike the two-track architecture used in AlphaFold and similar models, RoseTTAFoldNA includes a third track that directly processes 3D coordinates. By extending the definition of pre-defined frames to include nucleic acids, the model can predict the structures of both proteins, RNA, and DNA within a single unified system. This three-track design is expected to be effective because it allows information to flow between sequence data and spatial geometry throughout the network. By updating these representations together, the model aims to capture the complex spatial relationships between different types of molecules more accurately than previous methods.

AlphaFold3 builds upon the success of its innovative predecessor, AlphaFold2, but replaces the structure module with a new diffusion architecture. In this architecture, the network initializes the 3D space with a random distribution of atoms and gradually refines their positions into a physically realistic structure through multiple steps. This approach is a significant change from the previous version because it predicts individual atomic coordinates instead of relying on pre-defined frames for each residue. This new design provides great flexibility for modeling different types of molecules together because the model is not limited by fixed assumptions for each part of the structure. AlphaFold3 is able to predict 3D structures of complexes containing proteins, nucleic acids (DNA and RNA), small ligands, and ions. In response to this development, models with the same capabilities were published in rapid succession. The RoseTTAFold team released its own direct successor, RoseTTAFold All-Atom.^69^ Furthermore, because the code for AlphaFold3 was not immediately released after its publication, various models with an AlphaFold3-like architecture, such as Boltz-1,^70^ Chai-1,^71^ Protenix,^72^ and HelixFold3,^73^ were released.

The principal advantage of these generalist models is their versatility. They allow researchers to predict and study molecular interactions (for example between RNA and proteins or between proteins and small molecules) in combination with structure prediction. Previous methods required separate procedures for both structure prediction and docking, which has fundamental problems. For example, the reliability of docking is reduced when the predicted individual structures are inaccurate, and it is difficult to capture larger structural dynamics during docking. A unified framework that performs both structure prediction and docking can overcome these issues, providing new opportunities to study a wide range of molecular complexes.

Despite the advantages, this paradigm also presents drawbacks and new challenges. One important issue is the balance between specialization and generalization. Models trained on multiple types of biomolecules may not capture the subtle and unique structural characteristics of complex RNAs with numerous non-canonical interactions as accurately as RNA-specific models that are trained and optimized solely on RNA data. In addition, the predictive accuracy of these models is fundamentally constrained by the availability of structural data. The number of experimentally determined RNA and RNA-protein complex structures deposited in the PDB remains much smaller than the number of protein structures. This imbalance can introduce bias and may limit performance on novel RNA folds.

## RNA DESIGN

Similar to protein design, RNA design aims to produce RNA sequences that fold into a desired secondary structure; a tertiary structure; or have a desired function such as ribozymes, aptamers, riboswitches, and regulatory elements with tailor-made structural and functional properties.

### Conventional approaches

**Figure 3.**
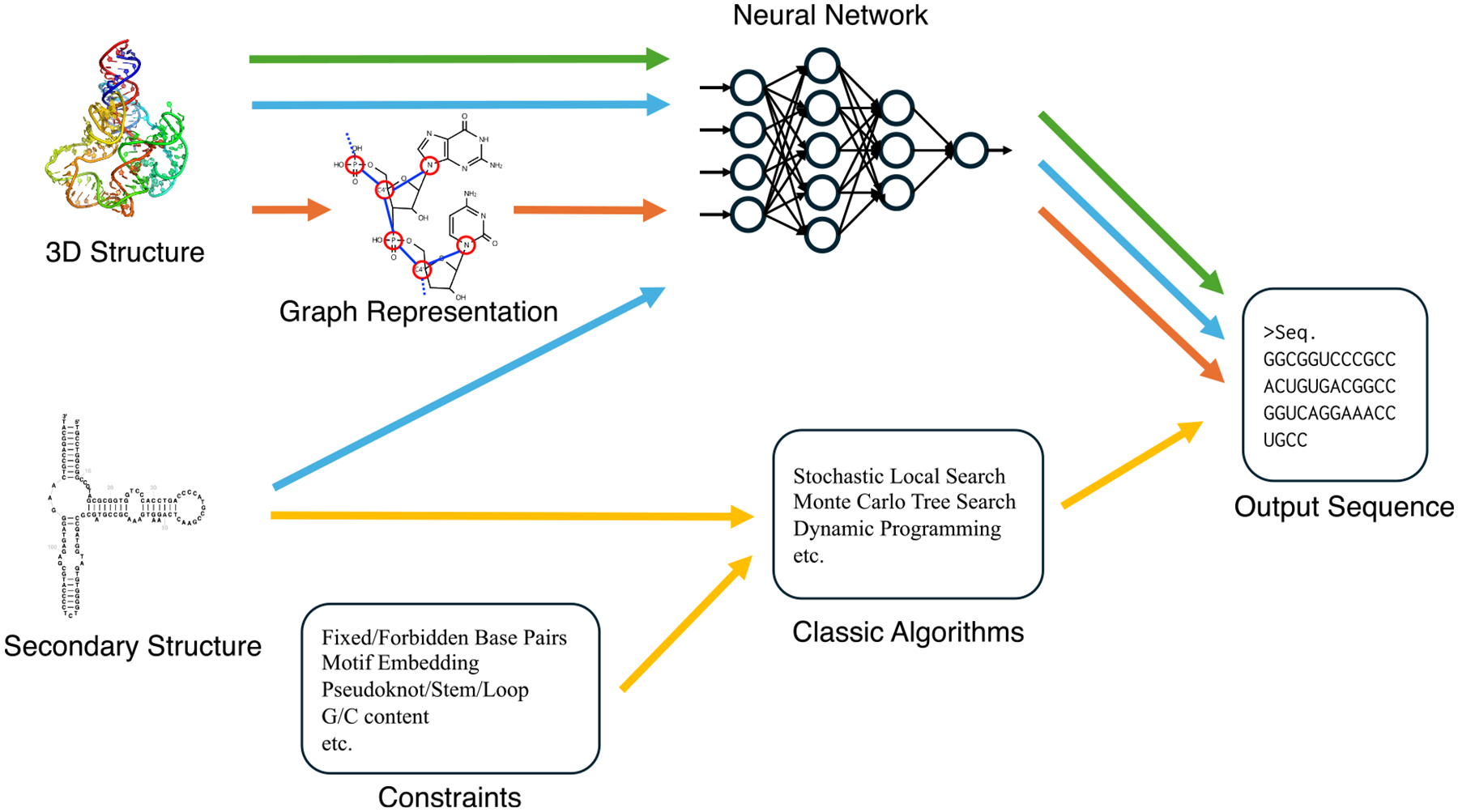
Overview of methods for RNA inverse folding Two main classes of approaches are illustrated: conventional algorithms (yellow arrows) compute candidate RNA sequences based on secondary structures and constraints. Deep-learning-based inverse folding approaches (green, blue, and orange arrows) predict RNA sequences from 3D structural inputs, with optional incorporation of secondary structure.

Early efforts in the 1990s and 2000s were primarily based on combinatorial and energy optimization approaches, aiming to identify RNA sequences that minimize the energy toward a target secondary structure. Methods in these years did not address design of tertiary structures of RNAs. Conventional methods relied on dynamic programming and stochastic search algorithms to solve the RNA inverse folding problem at the level of base pairing.^74–76^ The conventional inverse folding methods are represented by the yellow and red arrows in Figure 3.

RNAinverse (ViennaRNA)^74^ accepts a target secondary structure and an optional initial sequence as input. It begins with a random or a heuristically chosen sequence and iteratively mutates single bases or base pairs to reduce the structural distance to the target. This approach was applied to examples of the secondary structures of hairpins, internal loops, and short multi-loop structures. INFO-RNA^75^ takes a target secondary structure as input and uses a two-stage algorithm to generate desired sequences. It first generates an initial sequence using dynamic programming to minimize the energy of the target structure, then it refines it via a stochastic local sequence search. INFO-RNA was applied to sequences up to 1,975 nt. NUPACK^76^ generalizes RNA design to the secondary structure of multi-strand complexes that do not include pseudoknots.

After 2010, more advanced algorithms were proposed that support more user-specified constraints, enable the design of structures with pseudoknots, and explore a larger sequence search space. RNAiFold^77^ uses constraint programming to search for sequences that fold into a target secondary structure while also supporting consideration of user-specified constraints such as GC content, sequence motifs, or base-pairing rules. Using this approach, RNAiFold designed 54-nt *cis*-cleaving hammerhead ribozymes. Moreover, the algorithm was used to design a 166-nt synthetic RNA, which embeds a functional *cis*-cleaving hammer-head. To experimentally validate this computational design, the 166-nt RNA was synthesized and tested for self-cleavage activity. Monte Carlo Tree Search (MCTS)-RNA^78^ introduced a stochastic tree-search technique to improve exploration of sequence space. By using MCTS combined with local refinement, MCTS-RNA efficiently identifies longer sequences, which fold into target secondary structures, including pseudoknots. It can also consider GC content. It demonstrated capability of designing RNA sequences up to 348 nt. RAG-IF^79^ converts input secondary structure into planar, undirected, connected tree graphs, where loops and helices are modeled as vertices and edges. It identifies key residues for mutation by analyzing discrepancies between the designed and target topologies. A genetic algorithm then introduces mutations at these positions and iteratively refines the sequences to retain only the minimal set of essential changes.

### RNA origami

RNA origami was conceptually inspired by DNA origami,^80^ which originated in the early 1980s as an effort to design nucleotide sequences that fold into specified geometric shapes. Although its goals differ from those of RNA structure prediction and functional RNA design, RNA origami represents an earlier and independent line of nucleic-acid design focused on constructing geometrical nanostructures. RNA origami aims to design a single RNA strand that folds into a desired nanoscale shape through programmed intramolecular base-pairing and tertiary interactions. Unlike traditional multistranded assemblies, unimolecular RNA origami allows co-transcriptional folding directly during RNA synthesis, making it inherently compatible with genetic expression and *in vivo* applications.^81^ A comprehensive overview of RNA origami principles, design tools, and emerging applications is provided in the review by Poppleton et al.^80^

**Figure 4.**
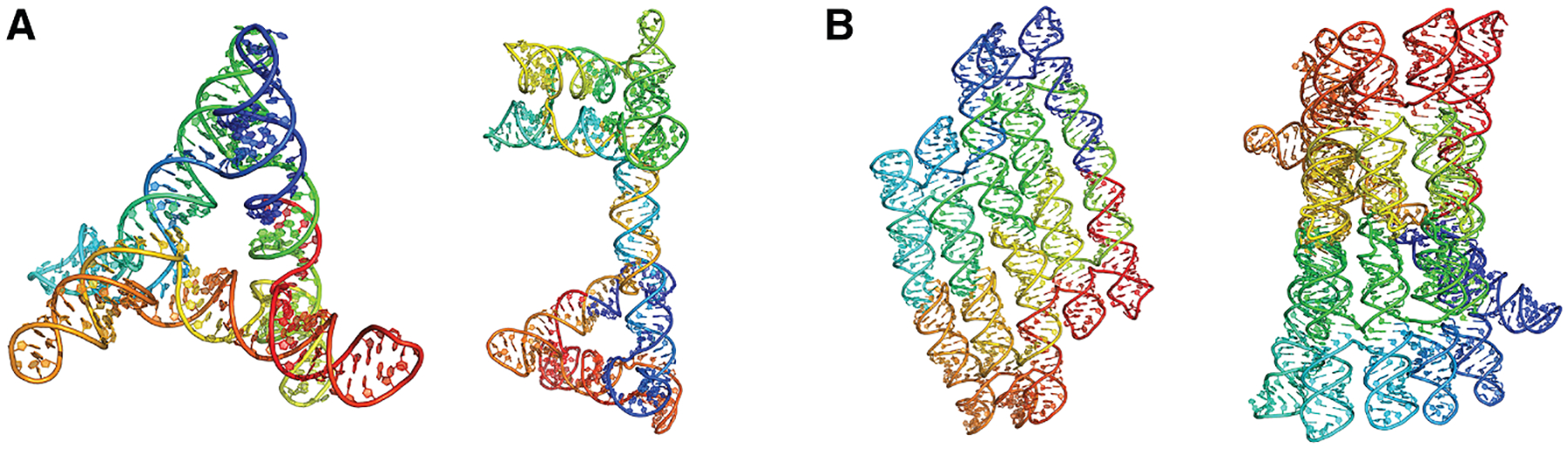
Single-strand RNA origami structures Sequences are colored from blue to red for the direction of 5′ end to 3′ end. These structures are all one RNA chain. (A) RNA origami structures designed manually by Vallina et al.^82^ (PDB: 8BTZ; total length, 238 nt. PDB: 8BU8; total length, 354 nt). (B) RNA origami structures designed by ROAD^83,84^ software (PDB: 7QDU; total length, 552 nt. PDB: 7PTK; total length, 720 nt).

Early work on single-stranded RNA origami demonstrated that long single-stranded RNA molecules, which span several kilo-bases, can be routed and folded into complex, knot-free architectures.^81,82^ Building on this advance, RNA Origami Automated Design (ROAD) provided the first automated computational platform for designing such scaffolds.^83^ First, ROAD uses RNAbuild to assemble modular 3D RNA motifs into a complete atomic-level scaffold model. Next, RNApath analyzes the co-transcriptional folding pathway, identifying potential topological barriers, such as premature pseudoknot formation, which could trap the strand during synthesis. Finally, the Revolvr sequence design engine optimizes the nucleotide sequence to satisfy multiple constraints simultaneously. Together, these steps enable ROAD to rationally design kilobase-scale RNA origami with placement of functional motifs such as aptamers or protein-binding domains. Examples of single-strand RNA origami structures are shown in Figure 4.

### Deep-learning approaches

Deep learning has also made substantial progress possible in RNA design. Particularly, it shifted RNA inverse folding from secondary-structure-based optimization to 3D-structure-conditioned generation. Deep-learning-based methods can learn complex sequence-structure relationships directly from a large dataset of tertiary RNA structures, enabling scalable and flexible design of structurally complex RNAs. These deep-learning-based approaches correspond to the green, blue, and orange arrows in Figure 3.

A representative example of this new paradigm is RhoDesign,^85^ a deep-generative structure-to-sequence model that formulates RNA inverse folding as a conditional generation problem. RhoDesign encodes the target RNA tertiary backbone using a geometric vector perceptron (GVP) and integrates these structural embeddings with secondary-structure contact maps, which are then processed by a transformer encoder-decoder to autoregressively generate RNA sequences. Trained end to end on a combination of experimentally resolved PDB structures and large-scale RhoFold-predicted RNA structures, the model learns a probabilistic mapping from RNA 3D geometry to sequence space and can generate sequence candidates that are consistent with the input structure. Represented by blue arrows in Figure 3, RhoDesign takes a target RNA 3D backbone as input and generates novel sequences (i.e., sequences that are very different from known RNAs) predicted to fold into the designed structure. When conditioned on the Mango-III aptamer structure (PDB: 6UP0), RhoDesign produced sequence candidates that are only 43%–65% identical to the known RNA but predicted to have similar structures to the target. The RMSD of the predicted structures to the target structure was 2.9–7.5 Å. Top candidates were synthesized for experimental validation. Experimental results confirmed that several generated sequences were functional.

**Figure 5.**
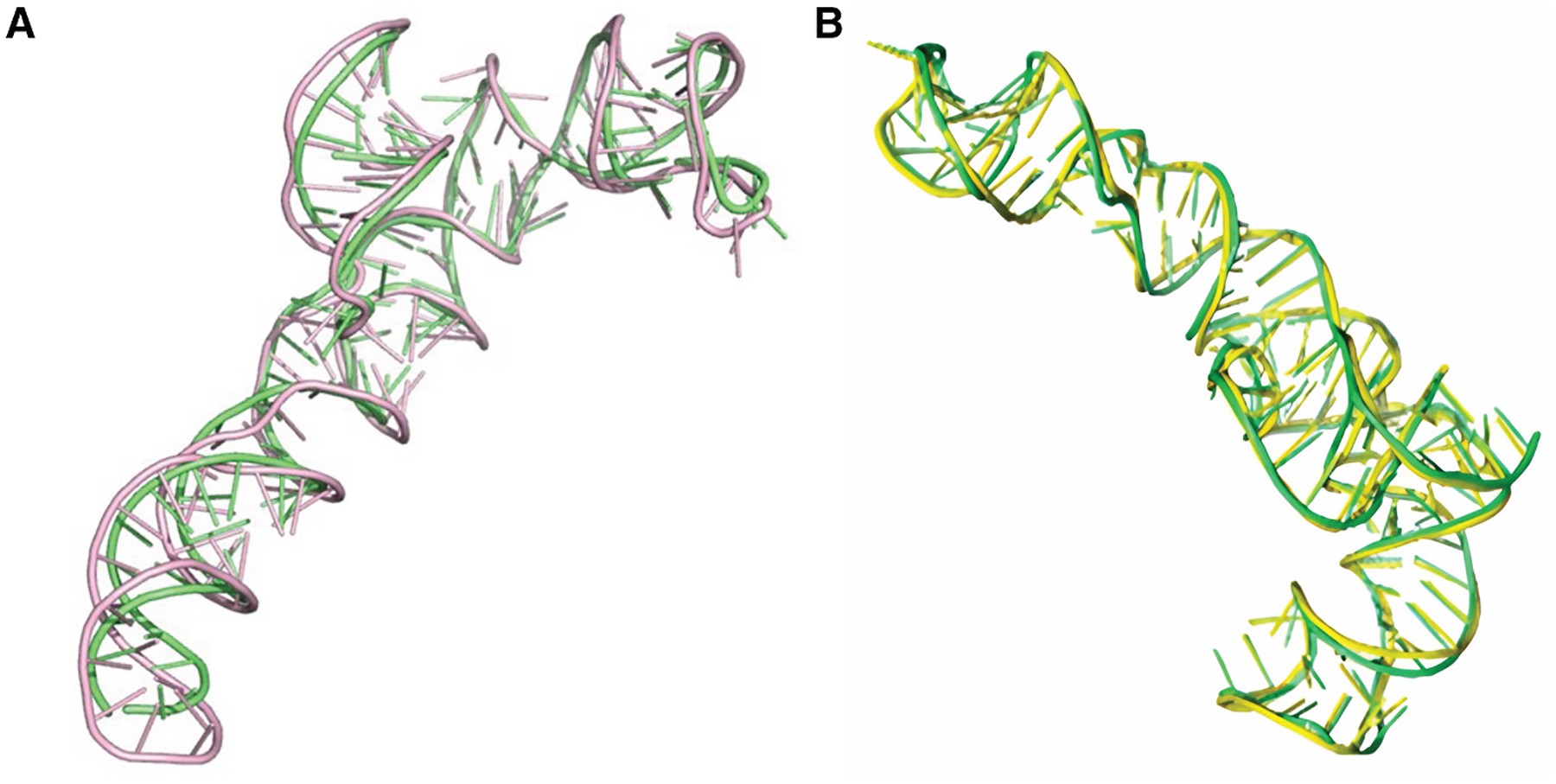
Examples of structures of designed RNA sequences by deep-learning methods (A) Predicted structure of a RiboDiffusion-designed sequence (green) and its target structure 6WQQ (pink). The structure was predicted by RhoFold. Length, 111 nt; TM score, 0.7542; sequence recovery rate, 0.5766. This figure was modified from Figure 4 of the RiboDiffusion paper with a permission under Creative Commons Attribution License. (B) Predicted structure of a RIdiffusion-designed sequence (green) and its target structure 5XXB (yellow). Length: 118 nt. RMSD: 1.26 Å. Recovery rate: 0.6780. This figure was modified from Figure 5 of the RIdiffusion paper with permission (American Chemical Society, 6154410161392).

Another recent development is RiboDiffusion,^86^ which formulates RNA inverse folding as a conditional generative diffusion process. In this framework, RNA sequences are represented as embeddings and progressively corrupted by Gaussian noise during training, while a neural network is trained to reverse this process and recover the original sequence conditioned on a fixed RNA 3D backbone (illustrated by green arrows in Figure 3). RiboDiffusion uses a GVP-graph neural network (GNN) structure module to encode geometric information and a transformer-based sequence module to capture intra-sequence dependencies. These two components are coupled through cross-attention, allowing the sequence denoiser at each diffusion step to condition on backbone-derived geometric embeddings. During generation, the learned reverse-diffusion process iteratively refines an initially random sequence embedding toward an RNA sequence consistent with the target backbone. By tuning sampling weights, this method allows balancing sequence recovery and diversity, supporting the generation of multiple structurally valid candidates. Its performance is strongest for medium-length RNAs (50–100 nt), while short RNAs remain challenging due to conformational flexibility and RNAs longer than 100 nt show gradual accuracy decay. Figure 5A shows an example of the structure of a RiboDiffusion-designed sequence (predicted by RhoFold) and its corresponding 3D target structure.

The most recent method RIdiffusion by Hou et al. introduced another type of diffusion model, called hyperbolic discrete diffusion for RNA inverse folding, which is suitable for representing the hierarchical nature of RNA 3D structure, from base pairing, secondary-structure elements, and remote pseudoknots to complex 3D shapes.^87^ The method is illustrated by orange arrows in Figure 3. RIdiffusion embeds structural features into a hyperbolic latent space using equivariant graph neural networks (HEGNNs), which can better capture subtle structural variations than conventional methods. Benchmark analyses show that RIdiffusion achieves competitive sequence recovery (i.e., the agreement of designed sequence to the sequence of the target RNA) across RNA families and evaluation splits, with the most consistent results observed for RNAs in the ranges of 50–100 nt and >100 nt. A sequence designed by RIdiffusion was predicted by RhoFold and compared with its 3D target structure in Figure 5B.

Parallel to advancements in standard RNA inverse folding, deep learning has also been applied to protein-conditioned RNA design to create molecules that bind specific protein targets. A representative method is RNAFlow,^88^ which uses flow matching, a generative deep-learning model (green arrows in Figure 5). RNAFlow receives a protein sequence and its backbone structure together with a noised RNA backbone that is the starting RNA conformation and outputs a designed RNA sequence and its predicted 3D structure. RNAFlow’s denoising network integrates two components: (1) a GVP-based inverse folding module (“Noise-to-Seq”) that predicts RNA sequences from protein-RNA complex geometry, and (2) a frozen RosettaFold2NA (RF2NA) structure predictor that folds the predicted sequence to provide structural supervision. During training, the Noise-to-Seq module is optimized using a flow-matching objective to predict denoised RNA sequences, with the RF2NA-predicted structures used to supervise the denoising process. During inference, the model iteratively updates the RNA backbone and sequence over a small number of flow steps, starting from a noisy initialization and producing a final RNA sequence together with its corresponding predicted structure. An example of designed RNA sequences by RNAFlow and the predicted structure by RF2NA is shown in Figure 7A.

**Figure 6.**
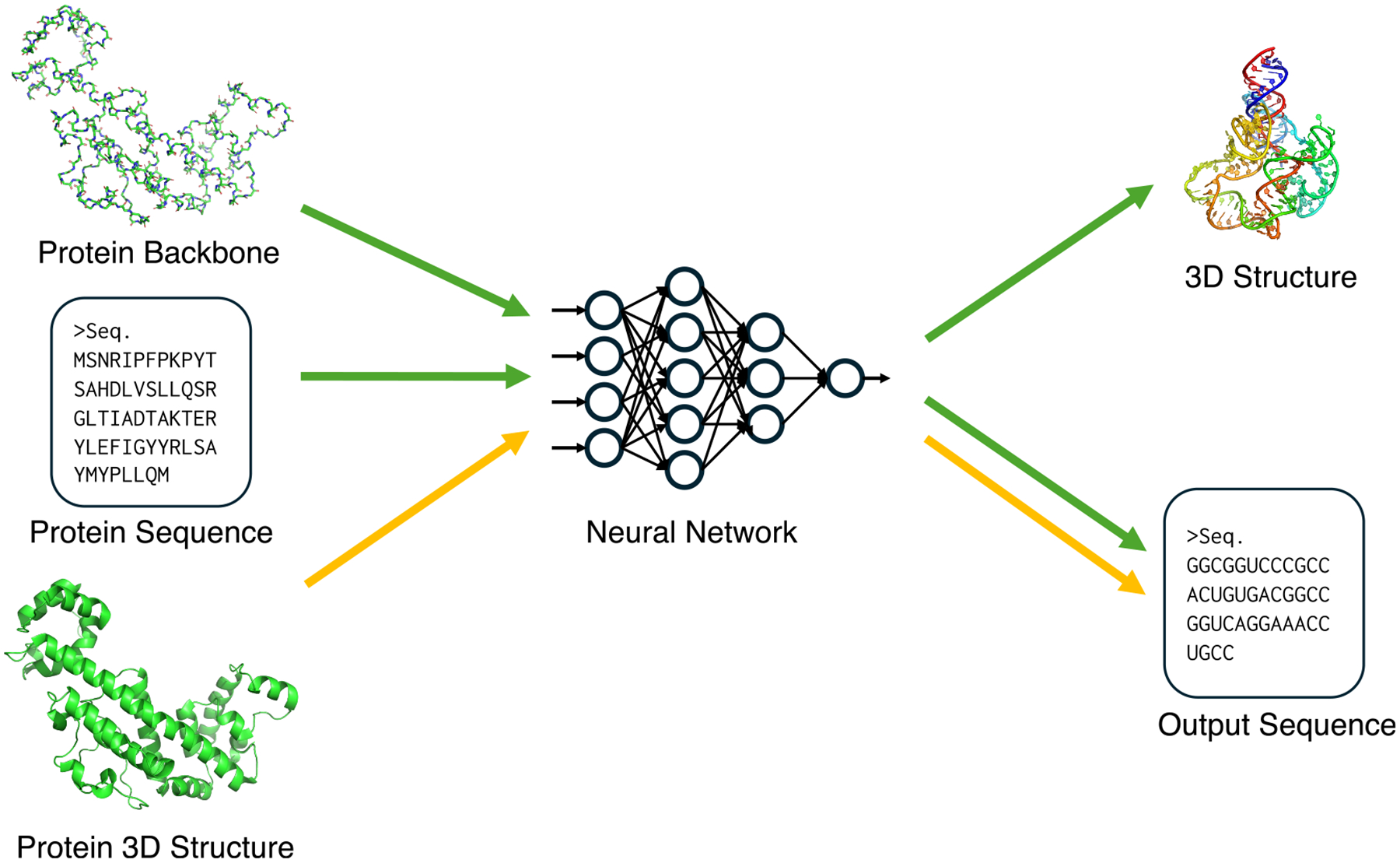
Deep-learning-based protein-binding RNA design methods Generative neural networks (RNAFlow and RNA-EFM, represented by green arrows) receive protein sequences and backbones to generate protein-binding RNA sequences and their 3D structures. Transformer-based method (RNA-BAnG, represented by yellow arrows) receives protein 3D structures to predict RNA sequences that bind to the target protein.

**Figure 7.**
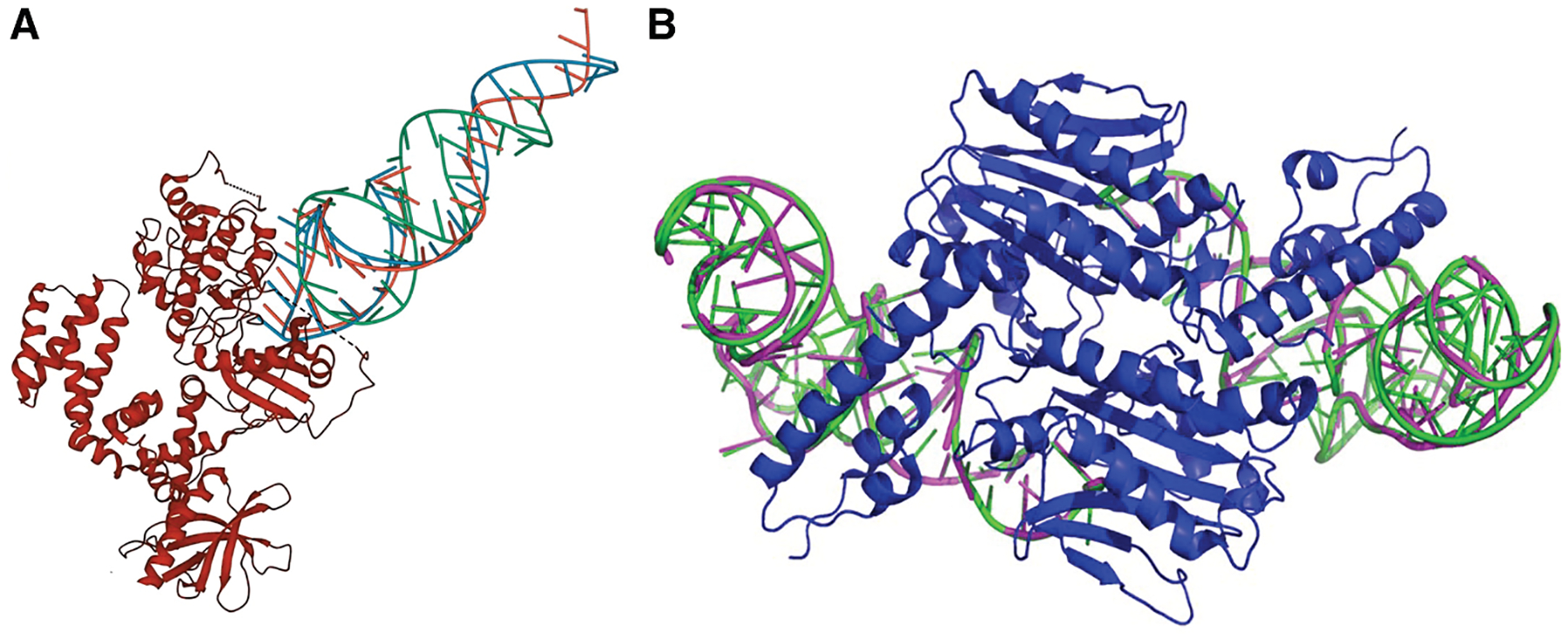
Examples of designed protein-binding RNAs by deep-learning methods Both structures are predicted by RosettaFold2NA for generated RNA sequences. (A) Predicted structures of RNA sequences (green, blue, and orange) that bind to GRK2 designed by RNAFlow. The RNA portion of the target structure 3UZS is not shown. RMSD, 7.09 Å; sequence length, 28 nt; sequence recovery rate, 0.54. This figure was modified from Figure 4 of the RNAFlow paper under the Creative Commons Attribution License. (B) Structure prediction of an RNA sequence designed by RNA-EFM (magenta) and its ground truth structure in PDB (PDB: 2ZNI) (green). RMSD, 2.93 Å; sequence length, 72 nt; sequence recovery rate, 0.54. This figure was modified from Figure 6 of the RNA-EFM paper under the Creative Commons Attribution License.

RNA-EFM^89^ is another protein-conditioned RNA sequence-structure co-design framework that jointly generates RNA sequences and 3D backbones given only a protein target as input (represented by green arrows in Figure 6). Similar to RNAFlow, it couples flow-matching-based inverse folding with a frozen RF2NA structure predictor to ensure geometry-aware sequence generation. In contrast to RNAFlow, which relies primarily on data-driven flow trajectories, RNA-EFM explicitly augments the flow-matching objective with an energy-based refinement stage that incorporates biophysical constraints during training and inference. RNA-EFM extends this strategy by introducing a physics-guided refinement objective that integrates Lennard-Jones potentials and sequence-derived free-energy constraints. This refinement objective aims to ensure that the designed RNA not only matches the intended geometry but also forms stable structures capable of maintaining protein-RNA interactions. An RNA sequence designed by RNA-EFM and predicted by RF2NA is shown in Figure 7B.

Instead of flow-matching-based generative networks, RNA-BAnG^90^ introduced a transformer-based approach for protein-conditioned RNA design (represented by yellow arrows in Figure 6). Rather than generating sequences autoregressively from one end, RNA-BAnG employs a bidirectional anchored generation strategy, which begins from an interaction motif located near the protein interface and expands the RNA sequence outward in both directions. This method reflects the observation that protein-binding RNAs often contain short functional cores embedded within larger structural contexts. Using geometric attention to encode the input protein structure, the model conditions RNA sequence generation on both local protein-RNA contacts and global protein sequence context.

Evaluation of RNA design methods is critical for assessing their effectiveness. Classic inverse folding approaches primarily focused on secondary-structure accuracy, comparing the predicted secondary structure of designed sequences to the target using metrics such as sensitivity, positive predictive value (PPV), and F1 score for base-pair prediction. These metrics reflect how well the designed sequence achieves the intended base-pairing pattern. In contrast, deep-learning-based methods have shifted toward tertiary structure evaluation, using metrics such as RMSD and TM score to quantify the similarity between the predicted 3D structure of the designed RNA and the target backbone.^85–87^ Additionally, sequence recovery rate, which is the fraction of nucleotides matching the native sequence, is widely reported across both paradigms. For protein-conditioned RNA design, in addition to recovery rate and structure accuracy, binding affinity prediction via deep-learning models is often used to evaluate whether the designed RNA forms functional interactions with the target protein.^90^ Together, these metrics provide a framework for benchmarking design algorithms and guiding improvements.

## CHALLENGES AND FUTURE DIRECTION OF THE FIELD

The advancement of deep learning has opened a new era in computational RNA structure prediction and RNA design. However, accurate prediction of RNAs remains a significant challenge, particularly for large RNAs or those with complex topologies. A major limitation arises from the scarcity of experimentally determined RNA structures available for training. Although deep learning is highly effective at generalizing from large datasets, its performance is constrained when data are limited. Because the number of high-resolution RNA structures is increasing only gradually, the development of techniques that can leverage additional data modalities will be essential to surpass the accuracy of current state-of-the-art models. Such approaches include the integration of chemical probing data, which provide local structural constraints, and low-resolution structural analysis methods that can validate global molecular architecture.

To partly cope with the scarcity of known RNA structures, most current deep-learning-based RNA structure prediction methods use an 80% sequence-identity threshold, which is significantly higher than the 25%–40% that is used in protein research, to split training and testing datasets. As similar RNAs would be included in both training and test sets (data leakage), it is observed that the accuracy of the methods decreases significantly when predicting structures of orphan RNAs or novel folds.^91,92^ Furthermore, this problem may not be solved simply by lowering the sequence-identity threshold because RNA molecules often maintain similar structural folds even when their sequences show very low similarity. Thus, relying on sequence-based criteria alone may be insufficient to ensure that datasets are truly independent. The recent CASP16 assessment highlighted these limitations by reporting that no predictions for previously unseen natural RNA structures achieved TM scores above 0.8.^15^ The RNA3DB framework has been proposed to address these issues.^93^ In addition to sequence homology, this approach considers structural homology by mapping RNA sequences to Rfam families^30^ to ensure that different members of the same structural family do not span across the divided datasets. The adoption of such refined datasets and evaluation methods will contribute to the development of more reliable and robust computational tools. Current models also generally struggle to reproduce complicated structural elements such as pseudoknots, non-canonical base pairs, and tertiary motifs such as A-minor interactions and G-quadraplex. These features were often missing or incorrectly modeled in most automated predictions. Predicting RNA multimers or RNA-protein interfaces is also challenging. Even when templates for individual components exist, assembling them into a biologically realistic complex with accurate interfaces is still difficult.

In RNA design, methods remain limited by the sequence length and structural complexity. Many methods achieve their strongest performance on short to medium-length RNAs (typically 50–100 nt) and show degraded accuracy for longer sequences that contain multiple tertiary motifs or long-range interactions.^85–87^ Protein-conditioned RNA design faces additional challenges, as current methods rely on imperfect structure prediction models and protein-RNA binding estimators during the design process.^88–90^

More fundamentally, current models are limited in their ability to capture the dynamic nature of RNA, as they typically generate a single static conformation. RNA molecules are inherently more flexible than proteins, and this conformational flexibility is often central to their biological functions. In the latest CASP16, this challenge was highlighted by a target focused on the dynamics of an RNA in solution, where most groups attempted to capture the dynamics using molecular dynamics (MD) simulations, with only limited use of machine-learning prediction models for this task.^94^ A key direction for future research will be the development of computational frameworks capable of accurately representing RNA dynamics and predicting conformational ensembles.

As the field of RNA 3D structure prediction continues to advance, it holds great promise for transforming our understanding of RNA biology and contributing to molecular medicine. The integration of advanced computational approaches with diverse experimental data is expected to improve both the accuracy and reliability of RNA structure prediction. These advancements will ultimately serve as a foundation for identifying novel RNA targets for therapeutic intervention, particularly in complex diseases in which RNA plays a central regulatory role.

## Figures and Tables

**Table 1.** Methods for RNA 3D structure prediction and design

Name	Category	Core principle/key technology	Input	Availability
3D structure prediction. Conventional methods				
ModeRNA	template-based	physics-based	template structures, alignment	code: https://genesilico.pl/moderna/download/ server: https://iimcb.genesilico.pl/modernaserver/
RNAbuilder	template-based	physics-based	template structures, constraints	code: https://simtk.org/frs/?group_id=359
Foldalign	threading	alignment with Sankoff’s algorithm	sequences	server: https://rth.dk/resources/foldalign/
LocARNA	threading	probabilistic alignment with Sankoff’s algorithm	sequences	server: https://rna.informatik.uni-freiburg.de/LocARNA
LaRA	threading	integer linear programming	sequences	code: https://github.com/seqan/lara
Vfold3D	fragment assembly	fragment database lookup	sequence, secondary structure	server: https://rna.physics.missouri.edu/vfold3D2/
RNAComposer	fragment assembly	fragment database lookup	sequence, secondary structure	server: https://rnacomposer.cs.put.poznan.pl/
FARFAR2	fragment assembly	fragment database, Monte Carlo sampling	sequence	code: https://rosettacommons.org/software/download/ server: https://rosie.rosettacommons.org/farfar2
SimRNA	coarse-grained simulation	Monte Carlo simulation, Simulated annealing	sequence	code: https://genesilico.pl/software/stand-alone/simrna server: https://genesilico.pl/SimRNAweb
IsRNA	coarse-grained simulation	coarse-grained MD	sequence	code & server: https://rna.physics.missouri.edu/IsRNA/index.html
RNAJP	coarse-grained simulation	coarse-grained MD (junction focused)	sequence	code: https://rna.physics.missouri.edu/RNAJP/index.html
BRiQ	knowledge-based potential	Monte Carlo sampling	sequence	code: https://github.com/Jian-Zhan/RNA-BRiQ
Vfold-Pipeline	hybrid, modular assembly	physics-based/templates	sequence	code & server: https://rna.physics.missouri.edu/vfoldPipeline/
3D structure prediction. Deep-learning methods				
DeepFoldRNA	MSA based (two stage)	MSA transformer	MSA, secondary structure	code: https://github.com/robpearc/DeepFoldRNA server: https://aideepmed.com/DeepFoldRNA/
trRosettaRNA	MSA based (two stage)	RNAformer	MSA, secondary structure	code: https://github.com/gjoni/trRosetta server: https://yanglab.qd.sdu.edu.cn/trRosettaRNA/
NuFold	MSA based (end to end)	Evoformer, structure module	MSA, secondary structure	code: https://github.com/kiharalab/NuFold GoogleColab: https://colab.research.google.com/github/kiharalab/nufold/blob/master/ColabNuFold.ipynb
RhoFold+	MSA based (end to end)	RNA language model, structure module	MSA	code: https://github.com/ml4bio/RhoFold server: https://proj.cse.cuhk.edu.hk/aihlab/RhoFold/
DRfold	MSA-free (hybrid)	RNA transformer	sequence, secondary structure	code: https://github.com/leeyang/DRfold server: https://aideepmed.com/DRfold/
DRfold2	MSA free (end to end)	RNA language model, RNA transformer, denoising structure module	sequence	code: https://github.com/leeyang/DRfold2 server: https://aideepmed.com/DRfold2/
RoseTTAFoldNA	generalized complex prediction	SE(3)-Transformer	MSA	code: https://github.com/uwipd/RoseTTAFold2NA
AlphaFold3	Generalized complex prediction	diffusion model	MSA	code: https://github.com/google-deepmind/alphafold3 server: https://alphafoldserver.com/
RoseTTAFold All-Atom	generalized complex prediction	SE(3)-Transformer	MSA	code: https://github.com/baker-laboratory/RoseTTAFold-All-Atom
Boltz-1	generalized complex prediction	diffusion model	MSA	code: https://github.com/jwohlwend/boltz
Chai-1	generalized complex prediction	diffusion model	MSA	code: https://github.com/chaidiscovery/chai-lab server: https://lab.chaidiscovery.com/
Protenix	generalized complex prediction	diffusion model	MSA	code: https://github.com/bytedance/Protenix server: https://protenix-server.com/
HelixFold3	generalized complex prediction	diffusion model	MSA	code: https://github.com/PaddlePaddle/PaddleHelix server: https://paddlehelix.baidu.com/app/all/helixfold3/forecast
Design methods 2D inverse folding				
RNAiFold	RNA inverse folding	heuristic adaptive walk	secondary structure, constraints	code: https://github.com/clotelab/RNAiFold
MCTS-RNA	RNA inverse folding	Monte Carlo tree search	secondary structure, constraints	code: https://github.com/tsudalab/MCTS-RNA
RAG-IF	*de novo* RNA design	graph-based topology design, genetic algorithm mutation	secondary structure, graph topology, constraints	code: https://github.com/Schlicklab/RAG-IF
Design methods 3D inverse folding				
RhoDesign	*de novo* RNA design	geometric deep learning	3D structure	code: https://github.com/ml4bio/RhoDesign
RiboDiffusion	RNA inverse folding	diffusion model	3D structure	code: https://github.com/ml4bio/RiboDiffusion
RIdiffusion	RNA inverse folding	diffusion model	3D structure	code: https://github.com/byternaAdmin/RIdiffusion
Design methods Protein-conditioned design				
RNAFlow	protein-binding RNA design	flow-matching model	protein 3D structure, protein sequence	code: https://github.com/divnori/rnaflow
RNA-EFM	protein-binding RNA design	flow-matching model	protein3D structure, protein sequence	code: https://github.com/abrarrahmanabir/RNA-EFM
RNA-BAnG	protein-binding RNA design	transformer model	protein3D structure	code: https://github.com/rsklypa/RNA-BAnG
